# Authors’ reply to “CO_2_-derived variables as surrogates for tissue perfusion and oxygenation”

**DOI:** 10.1016/j.aicoj.2025.100011

**Published:** 2026-01-16

**Authors:** Jihad Mallat, Jean-Louis Teboul, Daniel De Backer, Gustavo A. Ospina-Tascón

**Affiliations:** a Critical Care Division, Integrated Hospital Care Institute, Cleveland Clinic Abu Dhabi. Abu Dhabi, United Arab Emirates; b Cleveland Clinic Lerner College of Medicine of Case Western Reserve University, Cleveland, OH, United States of America; c Faculty of Medicine, University of Banja Luka. Banja Luka, Republic of Srpska, Bosnia and Herzegovina; d Service de Médecine Intensive-Réanimation, AP-HP, Hôpital de Bicêtre, DMU CORREVE, Université Paris-Saclay, Le Kremlin-Bicêtre, France; e Faculté de Médecine Paris-Saclay, Université Paris-Saclay, Le Kremlin-Bicêtre, Paris, France; f Department of Intensive Care, CHIREC Hospitals (Brussels and Braine-l'Alleud-Waterloo), Braine-l'Alleud, Belgium; g Université Libre de Bruxelles, Brussels, Belgium; h Department of Intensive Care, Fundación Valle del Lili, Cali, Colombia; i Translational Research Laboratory in Critical Care Medicine (TransLab-CCM), Universidad Icesi, Cali, Colombia

Dear Editor,

We thank Drs. Dubin and Pozo for their interest in our review [1], and appreciate the opportunity to clarify our perspective.

Drs. Dubin and Pozo question the reliability of venous-to-arterial PCO_2_ difference (Pv-aCO_2_) for assessing blood flow relative to CO₂ production (VCO_2_), citing its sensitivity to VCO_2_ and to dissociation-curve shifts [2]. As noted in our review [1], these determinants are well recognized. We agree that Pv-aCO_2_ should not be used to judge adequacy of oxygen delivery; however, because it depends on both flow and VCO_2_, Pv-aCO_2_ remains appropriate for assessing blood flow relative to CO_2_ clearance. Effects on the CO_2_-dissociation curve are usually modest compared with the impact of reduced blood flow, and numerous studies have shown that Pv-aCO_2_ rises when blood flow is low and insufficient to clear CO_2_, even when oxygen-derived indices remain unchanged [3].

In their endotoxemic shock model, Drs. Dubin and Pozo reported that fluid resuscitation narrowed Pv-aCO_2_ and the mesenteric-to-arterial PCO_2_ difference (Pmes-aCO_2_) as both cardiac output (CO) and mesenteric blood flow increased, while the mucosal-to-arterial PCO_2_ difference (Pt-aCO_2_) remained elevated, leading them to conclude that Pv-aCO_2_ does not track microcirculatory flow [2]. However, in another septic shock model, dobutamine induced parallel changes in Pt-aCO_2_, Pmes-aCO_2,_ and Pv-aCO_2_, and both Pt-aCO_2_ and Pmes-aCO_2_ correlated strongly with intestinal villi blood flow (R^2^ = 0.69 and 0.63), indicating that microcirculatory alterations can be reflected in tissue and venous CO_2_ accumulation [4]. Moreover, in septic shock patients, changes in sublingual microvascular flow were tracked by the sublingual-to-arterial PCO_2_ difference, even independently of CO in one study [1]. We did not intend to present Pv-aCO_2_ as a perfect microcirculatory marker, but elevated Pv-aCO_2_ can reasonably suggest microcirculatory impairment once CO is in the normal/high range, as illustrated in Figure 3 [1].

Drs. Dubin and Pozzo suggest a prominent role for the Haldane effect [2]. The evidence summarized in our review [1] shows that this effect minimally influences Pv-aCO_2_. Moreover, several studies in septic shock and chronic heart failure reported dobutamine-induced reductions in Pv-aCO_2_ despite increases in CO and venous oxygen saturation [1], indicating their findings may not be generalizable.

While the respiratory quotient (RQ) can exceed 1 in certain aerobic conditions (e.g., overfeeding), these are uncommon in critically ill patients, where an RQ > 1 is generally a reliable marker of anaerobic metabolism. A weak correlation between RQ and Pv-aCO_2_ and arterial-to-venous oxygen content difference (Pv-aCO_2_/Ca-vO_2_) does not diminish its clinical relevance, which has been associated with outcomes [1]. Although Drs. Dubin and Pozzo attribute the poor correlation to CO_2_-dissociation curve effects, they also reported no correlation between RQ and the venous-to-arterial CO_2_ content difference/Ca-vO_2_ [5], a variable unaffected by such shifts, weakening their explanation. We acknowledge that Pv-aCO_2_/Ca-vO_2_ performs poorly in extreme hemodilution, but in other experimental models and clinical studies, it has shown good reliability. It is not a universal marker of anaerobic metabolism, yet it remains useful in selected clinical situations. We view this ratio as a context-dependent integrative indicator rather than a strict aerobic–anaerobic discriminator. A persistently high Pv-aCO_2_/Ca-vO_2_, despite normalized oxygen delivery, may reflect altered CO_2_-dissociation shifts or regional hypoperfusion, physiologically relevant findings even if not equivalent to anaerobiosis.

We acknowledge that a recent single-center trial found no benefit of a CO_2_–oxygen–derived algorithm over standard care in achieving >10% lactate clearance [6]. This study-imposed assessment at baseline, 2 h, and 12 h, and did not allow for taking advantage of the very dynamic nature of Pv-aCO_2_ relative to lactate. Hence, this does not invalidate the underlying physiology; it simply underscores the limits of relying on a single target to guide therapy in a complex condition like shock. As noted in our manuscript, the proposed algorithm (Figure 3) is physiologically grounded but needs multicenter validation [1]. We believe that when Pv-aCO_2_ and Pv-aCO_2_/Ca-vO_2_ are assessed together with lactate, venous oxygen saturation, and other tissue-perfusion markers, they can still provide meaningful physiological insight. This approach was endorsed by the expert panel in the recent ESICM 2025 guidelines on circulatory shock and hemodynamic monitoring [7].

## CRediT authorship contribution statement

JM and GOT prepared the initial draft and finalized the manuscript. JLT and DDB critically reviewed the manuscript and provided critical feedback on the final draft. All authors have read and approved the final version of the manuscript.

## Consent for publication

Not applicable.

## Ethics approval and consent to participate

Not applicable.

## Funding

No funding.

## Availability of data and material

Not applicable.

## Declaration of competing interest

All authors declare no financial interests.
